# Neighborhood-level deprivation mediates racial and ethnic disparities in HCC diagnosis in Texas

**DOI:** 10.1097/HC9.0000000000000536

**Published:** 2024-10-17

**Authors:** Itunu O. Sokale, Aaron P. Thrift, Hashem B. El-Serag, Abiodun O. Oluyomi

**Affiliations:** 1 Section of Epidemiology and Population Sciences, Department of Medicine, Baylor College of Medicine, Houston, Texas, USA; 2 Dan L. Duncan Comprehensive Cancer Center, Baylor College of Medicine, Houston, Texas, USA; 3 Section of Gastroenterology and Hepatology, Department of Medicine, Baylor College of Medicine, Houston, Texas, USA; 4 Clinical Epidemiology and Comparative Effectiveness Program, Section of Health Services Research and Center for Innovations in Quality, Effectiveness and Safety (IQuESt), Department of Medicine, Baylor College of Medicine, Houston, Texas, USA; 5 Texas Medical Center Digestive Disease Center, Houston, Texas, USA; 6 Center for Precision Environmental Health, Baylor College of Medicine, Houston, Texas, USA

**Keywords:** HCC, liver cancer, minoritized neighborhoods, neighborhood deprivation, racial/ethnic disparities

## Abstract

**Background:**

Texas has the highest HCC rates in the United States, and the greatest burden is among Hispanics. Racial and ethnic disparities in HCC incidence have multiple underpinning factors. We conducted a mediation analysis to examine the role of neighborhood disadvantage (Area Deprivation Index) as a potential mediator of the association between neighborhood race and ethnicity distribution and neighborhood HCC case counts in Texas.

**Methods:**

The primary outcome measure was counts of new HCC diagnoses per census tract based on Texas Department of State Health Services Texas Cancer Registry data. The primary exposure of interest was the race and ethnicity-based Index of Concentration at the Extremes (non-Hispanic Black ICE or Hispanic ICE). We assessed Area Deprivation Index as a potential mediator of the association between Black/Hispanic ICE and HCC case counts. We adjusted the analyses for selected census tract characteristics.

**Results:**

We analyzed 4934 census tracts containing 13,632 new HCC diagnoses reported to Texas Cancer Registry between 2016 and 2020. Racial minority (Black/Hispanic ICE)-concentrated neighborhoods had a higher socioeconomic disadvantage. The results of the mediation analyses showed that compared to non-Hispanic White–concentrated census tracts, non-Hispanic Black–concentrated census tracts and Hispanic-concentrated census tracts had higher case counts of HCC (total effects: adjusted case count ratio: 1.03 [95% CI, 1.02–1.04] and adjusted case count ratio: 1.09 [95% CI, 1.08–1.10], respectively). Approximately 48% and 15% of the neighborhood-level disparity in HCC case counts were attributable to neighborhood socioeconomic disadvantage in Black and Hispanic minoritized neighborhoods, respectively.

**Conclusions:**

Neighborhood HCC case counts varied by neighborhood race and ethnicity distribution. The variations were partly explained by neighborhood deprivation, with a stronger effect among Black-concentrated census tracts.

## INTRODUCTION

HCC is a major public health problem in the United States. HCC incidence rates increased 3-fold between 1975 and 2014.^1^ There are racial and ethnic variations in HCC incidence and mortality rates in the United States,^2^ with Hispanics and non-Hispanic Blacks disproportionately affected compared with non-Hispanic Whites.^3^ Texas has the highest HCC rates in the United States, and the highest rates are among Hispanics.^4^ Reasons for the observed racial and ethnic disparities in HCC incidence and mortality are likely multifactorial, including variations in HCC risk factors’ prevalence, disparities in prevention, severity of the disease, treatment, and genetic predisposition.^5^,^6^ Growing scientific evidence suggests that residential neighborhood-level factors influence health behaviors and outcomes both directly and indirectly, independent of individual-level socioeconomic characteristics, lifestyle, and genetics.^7^,^8^ The neighborhood-level social determinants of health are defined by variables that describe the socioeconomic, sociocultural, and environmental characteristics of the neighborhood in which a person lives.^9^,^10^ In cancer research, studies have shown that racial and ethnic disparities can often be attributed to a range of neighborhood-level social determinants of health (ie, the environment in which a person lives, works, and ages).^11^

Neighborhood-level social determinants of health factors may have a significant impact on HCC risk factors and outcomes, and they may influence health care accessibility, health care quality, adherence to provider prescriptions, and overall mortality.^12^,^13^ Prior studies, including ours, have shown associations between HCC incidence and neighborhood-level factors (eg, higher proportions of the Black/African American population) and neighborhood deprivation.^14^ A study found that area-level disadvantage explained some of the variations in the stage at which individuals from different racial-ethnic groups were diagnosed with HCC.^15^ Other studies suggested independent associations between HCC incidence and neighborhood concentrations of underrepresented minority populations.^15^,^16^ Understanding the drivers (or mediators) of increased HCC case counts among racial-ethnic minority communities may provide opportunities to deliver effective interventions. Mediation analysis offers the opportunity to investigate the underlying mechanism or process by which exposure and outcome variables are related and how they influence each other through a mediator variable.^17^ Several cancer outcomes studies have performed mediation analyses where measures were ascertained at both the individual and neighborhood levels.^15^,^18^,^19^ The current study is novel not only because it includes a mediation analysis but specifically a neighborhood-level mediation analysis.

Area Deprivation Index (ADI) is a measure of neighborhood socioeconomic disadvantage, which can represent place-based stressors, incorporating important aspects of poverty, low educational attainment, substandard housing, and lack of employment opportunities.^20^,^21^ We hypothesized that the magnitude and significance of the neighborhood race and ethnicity-HCC case count associations would change significantly depending on ADI as the potential mediator. In the current analysis, we examined the role of the ADI as a mediator of the relationships between neighborhood race and ethnicity and HCC case counts in Texas.

## METHODS

Our study population and accompanying data were drawn from the Texas Department of State Health Services Texas Cancer Registry (TCR) and the US Census Bureau (US Census). The TCR, a Surveillance, Epidemiology, and End Results (SEER) Registry, is one of the largest US statewide population-based cancer registries.^22^ TCR uses active and passive surveillance systems to collect data on all cancers diagnosed among Texas residents. The database comprises demographic and clinical characteristics, including diagnosis date and type of cancer. Primary site and histology are coded according to the International Classification of Diseases for Oncology—Third Edition (ICD-O-3). We identified HCC cases diagnosed from 2016 to 2020 within the TCR data set using a combination of ICD-O-3 site code C22.0 and histology codes 8170–8175. We used the US Census Bureau (US Census) 2016–2020 American Community Survey (ACS) 5-Year Estimates to compute the neighborhood-level variables. The ACS is a nationwide survey that collects and produces information on the US population’s social, economic, housing, and demographic characteristics.^23^ The US Census Bureau summarizes ACS estimates to specific geographic levels, including the census tract. The census tract, with an optimum population of ~4000 residents or 1600 housing units, is a small and relatively permanent statistical subdivision of a county designed to be homogeneous in terms of population characteristics, economic status, and living conditions.^24^ The census tract was the unit of analysis, and all Texas census tracts (n = 5265) were considered for inclusion in our analysis. TCR reported 14,313 unique people with new HCC diagnoses in Texas between January 1, 2016, and December 31, 2020. Out of the 5265 census tracts in Texas, we excluded 331 from the analysis for the following reasons: 12 were water-only tracts, 31 were primarily nonresidential tracts (eg, airports), and 288 had no ADI values (eg, tracts with sparse population). After these exclusions, 4934 census tracts were included in the final analysis. The eligible census tracts contained 13,658 HCC cases. Of these, 13,632 (95.2%) classified as Hispanic, non-Hispanic Black, or non-Hispanic White were included in the final analysis.

### Measures and data sources

#### Outcome measure: Count of new HCC diagnoses

The research file from the TCR included location reference data values for each HCC diagnosis, including the longitude (*X*) and latitude (*Y*) coordinate points that represent a patient’s address at the time of diagnosis. We overlaid the *X* and *Y* data on top of the Texas census tract boundaries and summed the count of HCC cases per census tract from 2016 to 2020 (5 y).

#### Independent measure: Index of Concentration at the Extremes—ICE (Black ICE or Hispanic ICE)

Index of Concentration at the Extreme (ICE) was originally introduced by Masey in 2001,^25^ and popularized for population health monitoring by Krieger et al.^26^ In a critical review of methods for understanding how ecological context affects individual disadvantage, Masey argued for neighborhood-level measures that operate on a continuum from extreme disadvantage to extreme privilege, hence the ICE^25^

$${\mathrm{ICE}}_{i}=\left(A_{i}–\;P_{i}\right)/T_{i},$$

where *A*_*i*_ is the number of persons classified as privileged (ie, non-Hispanic White) in neighborhood *i*, *P*_*i*_ is the number of persons classified as disadvantaged (ie, non-Hispanic Black or Hispanic) in neighborhood *i*, and *T*_*i*_ is the population of privileged and disadvantaged persons combined in neighborhood *i*. Therefore, the ICE values range from −1 through 0 to +1 for each neighborhood, where a value of −1 denotes all persons in the neighborhood are classified as disadvantaged, while +1 means all persons are classified as privileged. Additional details on how ICE was calculated for the current study are provided in the Supplemental Material, http://links.lww.com/HC9/B54.

#### Potential mediator: ADI

The ADI is a composite measure of neighborhood socioeconomic disadvantage that is based on 17 US Census indicators from the following 4 categories: poverty, education, housing, and employment.^27^ We used Singh’s formula^27^,^28^ to compute ADI scores for all the census tracts in Texas. Data to compute the ADI were retrieved from the 2016–2020 ACS 5-Year Estimates. A detailed list of the indicators used to compute the ADI is shown in Supplemental Table S1, http://links.lww.com/HC9/B55. ADI quartiles were used in the descriptive analysis, whereas for the mediation analysis, the raw ADI scores were reclassified into a percentile classification such that a higher percentile classification represents more deprived tracts.

#### Covariates

We adjusted the mediation analysis for a few selected census tract-derived characteristics that are known to have significant associations with HCC case counts. Specifically, these included: current smokers,^29^ obesity,^30^ 55+-year-olds,^31^ and urban versus rural residency.^32^ Percentage of current tobacco use and percentage of obese were retrieved from the 2016 CDC PLACES database.^33^ Percentage of 55 years and older per census tract was retrieved from the 2016–2020 ACS 5-Year Estimates. For urban versus rural residency at diagnosis, we used the USDA 2010 rural-urban commuting area codes to designate a census tract as either urban (primary codes 1 and 2) or rural (primary codes ≥3).^34^

### Statistical analysis

We compared several neighborhood-level characteristics across the 4 classes of the ADI quartile classification. Chi-squared tests and independent *t* tests were used for these comparisons. Before the mediation analyses, we assessed the associations among the exposure, mediator, and outcome variables. Using Poisson regression models, we first examined the relationship between Black ICE or Hispanic ICE (exposure) and HCC case counts (outcome). Second, we assessed the relationship between the ADI (mediator) and HCC case counts. For these straightforward Poisson regression models (not mediation analysis), the total population was used as an offset term.^35^ Third, we used a generalized linear model to assess the relationship between the ICE measures (exposure) and the ADI (mediator).

#### Mediation model

Causal mediation analysis offers the opportunity to analyze the potential effects of a third variable—mediator (*M*) in a causal relationship between a predictor variable (*X*) and an outcome variable (*Y*).^17^ The PROC CAUSALMED in SAS software uses a counterfactual framework that fits generalized linear models with normal, binary, negative binomial, or Poisson distribution to estimate the causal mediation effects.^36^ After summarizing the HCC cases into census tract boundaries, our analytic data set was highly skewed. To address the evidence of overdispersion observed in our data set—the variance of the dependent variable is greater than the mean—we used a mediation analysis procedure that could fit Poisson distributed data. Of note, the census tract population would be used as the offset term for each tract in a straightforward Poisson regression model to estimate the number of HCC cases per capita given the explanatory variables included in the model^35^,^37^; however, such offset function is absent for the Poisson-based PROC CAUSALMED mediation analysis. Consequently, our Poisson regression (without offset term) used the census tract total population as a control variable. For the mediation analyses, the census tract total population was reclassified into 4 classes based on the Jenks optimization method. Jenks minimizes variation within classes and maximizes variation between classes, such that census tracts that fall inside the same class are statistically more similar to each other than those outside of that class.^38^,^39^ Eventually, we calculated the case count ratio and 95% CIs per decile increase in the ICE measures.

**FIGURE 1:**
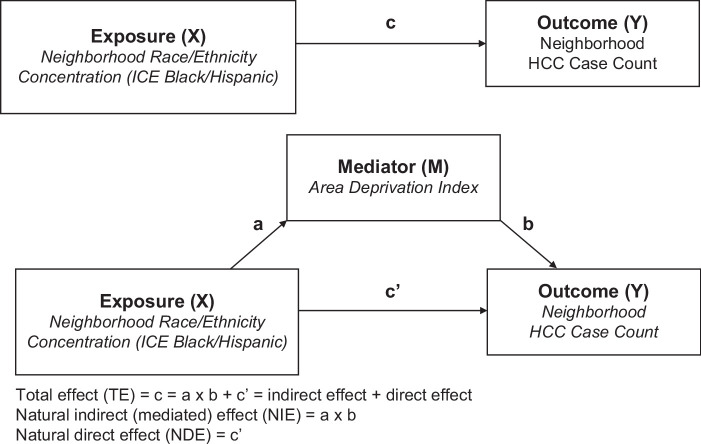
Causal mediation analysis model. Abbreviation: ICE, index of concentration at the extremes.

**FIGURE 2:**
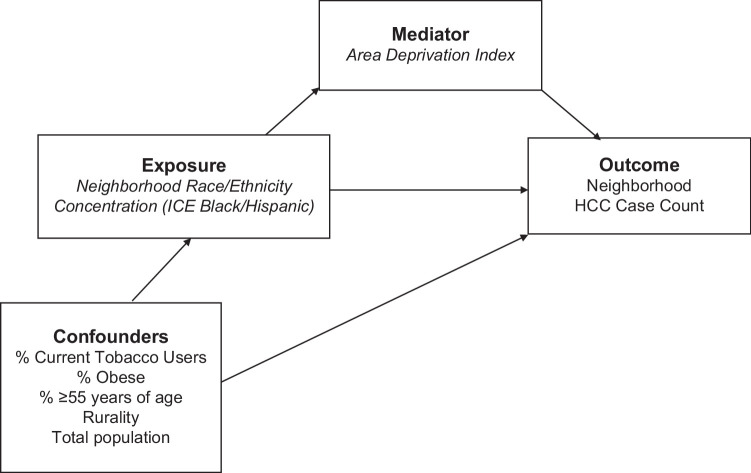
Components of the counterfactual framework–based mediation model. Abbreviation: ICE, index of concentration at the extremes.

The mediation model for this analysis is shown in Figures 1 and 2. Black ICE or Hispanic ICE represent the exposure (*X*), HCC case count per census tract was the outcome (*Y*), and ADI was the mediator (M). The potential effect of non-Hispanic Black–concentrated tracts or Hispanic-concentrated tracts on HCC case counts was considered the total effect (TE) (*c*). The effect of non-Hispanic Black–concentrated tracts or Hispanic-concentrated tracts on HCC case counts due to the ADI, that is, the mediator-adjusted effect, was considered the natural direct effect (*c*ʹ). The combination of the effect of race and ethnicity concentration on ADI (*a*) and the effect of ADI on HCC case counts (*b*) was the mediated effect or the natural indirect effect (*ab*). A mediation effect was present when the TE (*c*) and indirect effect (*ab*) were statistically significant, and the direct effect (*c*′) was smaller than the TE (*c*). Of note, the mediation analysis also produces the proportion mediated (PM), which provides “an estimate of the extent to which the total effect is accounted for by the pathway through the mediating variable,” and ranges from 0% (no mediation) to 100% (entirely mediated through ADI).^40^ We adjusted the mediation analysis for selected covariates that may moderate the magnitude of the mediating effects.

## RESULTS

Census tracts with higher disadvantage (ADI quartile 4) were found to have higher Black ICE and Hispanic ICE concentrations, and higher percentage of obese and percentage of smoking tracts (see Table 1 for details). As expected, we found evidence of a positive association between Black ICE (decile increase) and HCC case counts (incidence rate ratio = 1.05, 95% CI, 1.05–1.06) and Hispanic ICE (decile increase) and HCC case counts (incidence rate ratio = 1.08, 95% CI, 1.08–1.09). Also, a decile increase in Black ICE and Hispanic ICE increased ADI percentile scoring by a factor of 1.05 (95% CI, 1.05–1.06) and 1.06 (95% CI, 1.05–1.06), respectively. Last, there was also a positive association between ADI (percentile increase) and HCC case counts (incidence rate ratio = 1.10, 95% CI, 1.10–1.10).

**TABLE 1:** Neighborhood-level characteristics across ADI quartile classification

	n (%)				
	Quartile 1 (N = 1233)			Quartile 4 (N = 1233)	
Census tracts	Least deprived	Quartile 2 (N = 1234)	Quartile 3 (N = 1234)	Most deprived	*p*
ADI; Mean (Min, Max)	89 (−45.2, 103.4)	107.5 (103.4, 110.6)	113.1 (110.6, 115.3)	117.6 (115.3, 122.6)	
Black ICE					<0.001
Quartile 1	564 (45.7)	355 (28.8)	243 (19.7)	71 (5.8)	
Quartile 2	437 (35.4)	359 (29.1)	296 (24.0)	142 (11.5)	
Quartile 3	183 (14.8)	338 (27.4)	371 (30.1)	342 (27.7)	
Quartile 4	49 (4.0)	182 (14.7)	324 (26.3)	678 (55.0)	
Hispanic ICE					<0.001
Quartile 1	573 (46.5)	345 (28.0)	242 (19.6)	73 (5.9)	
Quartile 2	474 (38.4)	351 (28.4)	272 (22.0)	137 (11.1)	
Quartile 3	166 (13.5)	405 (32.8)	347 (28.1)	316 (25.6)	
Quartile 4	20 (1.6)	133 (10.8)	373 (30.2)	707 (57.3)	
% Tract pop. ≥55 y.o.					<0.001
Quartile 1	285 (23.1)	275 (22.3)	333 (27.0)	339 (27.5)	
Quartile 2	256 (20.8)	298 (24.1)	316 (25.6)	365 (29.6)	
Quartile 3	308 (25.0)	318 (25.8)	291 (23.6)	316 (25.6)	
Quartile 4	384 (31.1)	343 (27.8)	294 (23.8)	213 (17.3)	
% Tract pop. obese					<0.001
Quartile 1	877 (71.1)	284 (23.0)	59 (4.8)	15 (1.2)	
Quartile 2	306 (24.8)	566 (45.9)	294 (23.8)	56 (4.5)	
Quartile 3	42 (3.4)	333 (27.0)	599 (48.5)	278 (22.5)	
Quartile 4	8 (0.6)	51 (4.1)	282 (22.9)	884 (71.7)	
% Tract pop. smoking					<0.001
Quartile 1	936 (75.9)	238 (19.3)	35 (2.8)	6 (0.5)	
Quartile 2	250 (20.3)	600 (48.6)	300 (24.3)	119 (9.7)	
Quartile 3	36 (2.9)	318 (25.8)	542 (43.9)	336 (27.3)	
Quartile 4	11 (0.9)	78 (6.3)	357 (28.9)	772 (62.6)	
Tract urban vs. rural					<0.001
Urban	1225 (99.4)	1160 (94)	1071 (86.8)	1095 (88.8)	
Rural	8 (0.6)	74 (6)	163 (13.2)	138 (11.2)	

*Note*: The quartile classification of ADI was only for descriptive analysis.

Abbreviations: ADI: area deprivation index; ICE, index of concentration at the extremes.

The results of the mediation analysis are presented in Table 2. For each decile increase in Black ICE, the potential effect of non-Hispanic Black–concentrated tracts on 5-year HCC case counts (TE) was 1.03 (95% CI, 1.02–1.04), the effect of non-Hispanic Black–concentrated tracts on HCC case counts due to the ADI (natural direct effect) was 1.01 (95% CI, 1.01–1.02), and the mediated effect of ADI on HCC case counts (natural indirect effect) was 1.01 (95% CI, 1.01–1.02). The estimated extent of the TE accounted for by the pathway through the mediating variable (PM) was 48.0% (30.1%–65.8%), meaning that 48% of the TE of non-Hispanic Black–concentrated tracts on the 5-year HCC case counts was explained by ADI. In addition, for each decile increase in Hispanic ICE, the potential effect of Hispanic-concentrated tracts on 5-year HCC case counts (TE) was 1.09 (95% CI, 1.08–1.10), the effect of Hispanic-concentrated tracts on HCC case counts due to the ADI (natural direct effect) was 1.08 (95% CI, 1.06–1.09), and the mediated effect of ADI on HCC case counts (natural indirect effect) was 1.01 (95% CI, 1.01–1.02). The estimated extent of the TE accounted for by the pathway through the mediating variable (PM) was 15.0% (8.9%–21.0%), suggesting that 15% of the TE of Hispanic-concentrated tracts on the 5-year HCC case counts operated through the ADI.

**TABLE 2:** Mediation effects of ADI on the association between race and ethnicity and HCC case counts in Texas

	Causal effects models^a^		
	Adjusted case count ratio (95% CI)	*p*	Proportion mediated (95% CI)^b^
Non-Hispanic Black–concentrated neighborhoods vs. non-Hispanic White–concentrated neighborhoods			48.0 (30.1–65.8)
TE^c^	1.03 (1.02–1.04)	<0.001	
NDE^d^	1.01 (1.01–1.02)	0.003	
NIE^e^	1.01 (1.01–1.02)	<0.001	
Hispanic-concentrated neighborhoods vs. non-Hispanic White–concentrated neighborhoods			15.0 (8.9–21.0)
TE^c^	1.09 (1.08–1.10)	<0.001	
NDE^d^	1.08 (1.06–1.09)	<0.001	
NIE^e^	1.01 (1.01–1.02)	<0.001	

^a^ Models included an interaction term between race and ethnicity and ADI, and adjusted for percent individual currently smoking, percent obese, percent population 55 years or older, and rurality in each census tract.

^b^ *Proportion mediated* (PM) is an estimate of the extent to which the total effect is accounted for by the pathway through the mediating variable.

^c^ *Total Effect* (TE) is the potential effect of non-Hispanic Black–concentrated tracts or Hispanic-concentrated tracts on HCC case counts.

^d^ *Natural Direct Effect* (NDE) is the effect of non-Hispanic Black–concentrated tracts or Hispanic-concentrated tracts on HCC case counts due to the ADI.

^e^ *Natural Indirect Effect* (NIE) is the combination of the effect of race and ethnicity concentration on ADI and the effect of ADI on HCC case counts.

Abbreviation: ADI, area deprivation index.

## DISCUSSION

In this novel neighborhood-level mediation analysis, we found that neighborhood socioeconomic deprivation (ADI) substantially mediated neighborhood racial and ethnic disparity in HCC case counts. As expected, HCC case counts in census tracts varied by race and ethnicity with disproportionately higher HCC case counts in non-Hispanic Black–concentrated and Hispanic-concentrated census tracts than non-Hispanic White–concentrated census tracts. ADI explained almost half (48%) of the elevated HCC case counts in non-Hispanic Black–concentrated census tracts, while it explained 15% of the HCC burden in Hispanic-concentrated census tracts. These findings emphasize the critical role of neighborhood disadvantage on HCC risk and racial-ethnic disparities in HCC.

Prior studies have demonstrated the role of “place” in health disparities.^41^,^42^ Ortiz et al^43^ observed strong associations between neighborhood-level socioeconomic variables and high-risk liver cancer clusters in Pennsylvania, USA. A Louisiana-based study reported associations between living in most socioeconomically deprived neighborhoods and high HCC incidence^44^ and poor HCC survival.^45^ In each of these analyses, a measure of race and ethnicity was assessed at the individual level, whereas the measure of socioeconomic disadvantage was assessed at the area level. In our previous work that used factors exclusively measured at the neighborhood level, we found that higher neighborhood deprivation and the proportion of census tract residents that were non-Hispanic Black or Hispanic were independently associated with higher HCC incidence.^14^

The current study contributes to ongoing research that aims to address the question of how much neighborhood deprivation influences the relationship between race and ethnicity and cancer burden. The findings offer researchers and policymakers the opportunity to appreciate the proportion of the association between race and ethnicity and the cancer burden that remains unexplained by the neighborhood disadvantage. We have previously reported evidence of mediating effects of neighborhood disadvantage on the link between race and ethnicity and the stage at diagnosis for HCC^15^ and cervical cancer.^19^ Others have reported that area-level disadvantage significantly mediated race and ethnicity disparities in childhood cancer survivorship.^46^ Of note, the cancer burdens examined in the listed mediation analyses are postdiagnosis outcomes, alongside the race and ethnicity measures, at the individual patient level. In the current mediation analysis, the cancer burden was measured as incident HCC case counts (not a postdiagnosis outcome) assessed at the neighborhood level (not the individual level). Also, the race and ethnicity measures (exposure), the ADI (mediator), and the covariates included in the models were all assessed at the neighborhood level. With all measures resulting from neighborhood-level attributes, our findings are novel and elevate the importance of area-level characteristics as potent risk factors for HCC as a population health burden, and simultaneously offer the opportunity to target and resolve these modifiable area-level characteristics.

In the current study, the mediating effect of ADI was especially pronounced for the association of HCC case counts and non-Hispanic Black–concentrated tracts than Hispanic-concentrated tracts. In a previous analysis, we found that higher neighborhood-level disadvantage (ADI) was associated with a lower likelihood of being diagnosed with local HCC, and that ADI had a greater mediating effect on the race and ethnicity and HCC stage association when Hispanic individuals were compared with non-Hispanic White individuals (12.6%) than when non-Hispanic Black individuals were compared with non-Hispanic White individuals (2.3%).^15^ In that analysis, we suggested that certain non-socioeconomic factors may be of greater importance among non-Hispanic Blacks compared with Hispanics in terms of HCC diagnosis.^47^,^48^,^49^ The exposure-mediator-outcome interplay may (i) operate differently when assessed with individual-level measures versus exclusively with neighborhood-level measures, or (ii) yield varying results based on whether the outcome is diagnosis stage versus incident case counts. Overall, future research is needed to clarify differences in the roles that neighborhood disadvantage may play in race and ethnicity and HCC association.

The strengths of the current study include using population-based data over multiple years (2016–2020), and high-quality data that meet international standards. We analyzed all variables at equal levels (neighborhood levels), reducing the possibility of bias that may be introduced by mixing individual and place variables. Nevertheless, this study has limitations. First, although ADI is a widely used and validated comprehensive composite measure of neighborhood deprivation,^27^ it may not capture all dimensions of social disadvantage. Second, we cannot rule out the possibility of misclassification of the ICE (Black ICE and Hispanic ICE), which quantifies the concentration of the non-Hispanic Black (or Hispanic) population within a census tract. Another important limitation is that the COVID-19 pandemic caused significant disruptions in reporting to some cancer registries. Some analyses reported significant declines in incidence rates, particularly among screen-detectable cancers, at the initial stage of the pandemic, while rate changes in other cancer sites were not significant.^50^ Last, while our analyses have adjusted for some HCC risk factors, such as area-level measures of obesity and smoking, residual confounding remains related to HCV and HBV infection and alcohol-associated liver disease.

The current study used causal mediation analysis models that controlled for potential confounders, complements previous individual-level and neighborhood-level studies, and introduces a unique dimension offered by an all-round ecologic-level mediation analysis of the associations between exposures and outcomes and potential mediators. The findings highlight factors to consider in area-level interventions for HCC or to measure in individual patients (eg, stress biomarkers). Overall, our findings suggest that neighborhood-level disadvantage partially explained the associations between neighborhood-level racial and ethnic differences and HCC case counts. Therefore, interventions improving area-level socioeconomic status, particularly for neighborhoods with higher concentrations of racial and ethnic minority populations, could mitigate geographic disparities in HCC incidence.

## Supplementary Material

## Data Availability

The data sets used and/or analyzed during the current study are available from the corresponding author upon reasonable request. Itunu O. Sokale: methodology, formal analysis, investigation, data curation, writing—original draft, writing—review and editing, and visualization. Aaron P. Thrift: conceptualization, methodology, investigation, data curation, writing—review and editing, supervision, and funding acquisition. Hashem B. El-Serag: conceptualization, methodology, investigation, resources, writing—review and editing, supervision, and funding acquisition. Abiodun O. Oluyomi: conceptualization, methodology, formal analysis, investigation, resources, data curation, writing—original draft, writing—review and editing, visualization, and supervision.

## References

[1] Altekruse SF Henley SJ Cucinelli JE McGlynn KA . Changing hepatocellular carcinoma incidence and liver cancer mortality rates in the United States. Am J Gastroenterol. 2014;109:542. 24513805 10.1038/ajg.2014.11 PMC4148914

[2] Islami F Miller KD Siegel RL Fedewa SA Ward EM Jemal A . Disparities in liver cancer occurrence in the United States by race/ethnicity and state. CA Cancer J Clin. 2017;67:273–289. 28586094 10.3322/caac.21402

[3] White DL Thrift AP Kanwal F Davila J El-Serag HB . Incidence of hepatocellular carcinoma in all 50 United States, from 2000 through 2012. Gastroenterology. 2017;152:812–820.e5. 27889576 10.1053/j.gastro.2016.11.020 PMC5346030

[4] El-Serag HB Sardell R Thrift AP Kanwal F Miller P . Texas has the highest hepatocellular carcinoma incidence rates in the USA. Dig Dis Sci. 2021;66:912–916. 32303951 10.1007/s10620-020-06231-4

[5] Ge D Fellay J Thompson AJ Simon JS Shianna KV Urban TJ . Genetic variation in IL28B predicts hepatitis C treatment-induced viral clearance. Nature. 2009;461:399–401. 19684573 10.1038/nature08309

[6] Tian C Stokowski RP Kershenobich D Ballinger DG Hinds DA . Variant in PNPLA3 is associated with alcoholic liver disease. Nat Genet. 2010;42:21. 19946271 10.1038/ng.488

[7] Adler NE Rehkopf DH . US disparities in health: Descriptions, causes, and mechanisms. Annu Rev Public Health. 2008;29:235–252. 18031225 10.1146/annurev.publhealth.29.020907.090852

[8] Robert SA . Socioeconomic position and health: The independent contribution of community socioeconomic context. Annu Rev Sociol. 1999;25:489–516.

[9] Zeigler-Johnson CM Tierney A Rebbeck TR Rundle A . Prostate cancer severity associations with neighborhood deprivation. Prostate Cancer. 2011;2011:846263. 22111000 10.1155/2011/846263 PMC3195845

[10] Diez Roux AV Mair C . Neighborhoods and health. Annals of the New York academy of sciences. 2010;1186:125–145. 10.1111/j.1749-6632.2009.05333.x 20201871

[11] Christodouleas JP Baumann BC He J Hwang WT Tucker KN Bekelman JE . Optimizing bladder cancer locoregional failure risk stratification after radical cystectomy using SWOG 8710. Cancer. 2014;120:1272–1280. 24390799 10.1002/cncr.28544

[12] Shebl FM Capo-Ramos DE Graubard BI McGlynn KA Altekruse SF . Socioeconomic status and hepatocellular carcinoma in the United States. Cancer Epidemiol Biomarkers Prev. 2012;21:1330–1335. 22669949 10.1158/1055-9965.EPI-12-0124 PMC3647693

[13] Ruffolo LI Zambrano D Dale BS Nimmagadda SV Hack M Gaba H . Inferior survival is associated with socioeconomic deprivation in hepatocellular carcinoma. J Surg Res. 2022;279:228–239. 35792450 10.1016/j.jss.2022.05.035

[14] Oluyomi AO El-Serag HB Olayode A Thrift AP . Neighborhood-level factors contribute to disparities in hepatocellular carcinoma incidence in Texas. Clin Gastroenterol Hepatol. 2023;21:1314–1322. e5. 35933074 10.1016/j.cgh.2022.06.031 PMC9898456

[15] Oluyomi AO Mohammadi KA El-Serag HB Thrift AP . Mediating effects of neighborhood-level socioeconomic deprivation on the association between race/ethnicity and advanced hepatocellular carcinoma. Cancer Epidemiol Biomarkers Prev. 2022;31:1402–1409. 35314860 10.1158/1055-9965.EPI-21-1396 PMC9661396

[16] Chang ET Yang J Alfaro-Velcamp T So SK Glaser SL Gomez SL . Disparities in liver cancer incidence by nativity, acculturation, and socioeconomic status in California Hispanics and Asians. Cancer Epidemiol Biomarkers Prev. 2010;19:3106–3118. 20940276 10.1158/1055-9965.EPI-10-0863 PMC3005535

[17] Valeri L VanderWeele TJ . Mediation analysis allowing for exposure-mediator interactions and causal interpretation: Theoretical assumptions and implementation with SAS and SPSS macros. Psychol Methods. 2013;18:137. 23379553 10.1037/a0031034 PMC3659198

[18] Moss JL Liu B Feuer EJ . Urban/rural differences in breast and cervical cancer incidence: The mediating roles of socioeconomic status and provider density. Womens Health Issues. 2017;27:683–691. 29108988 10.1016/j.whi.2017.09.008 PMC5694385

[19] Sokale IO Oluyomi AO Montealegre JR Thrift AP . Racial/ethnic disparities in cervical cancer stage at diagnosis: Mediating effects of neighborhood-level socioeconomic deprivation. Cancer Epidemiol Biomarkers Prev. 2023;32:818–824. 37067295 10.1158/1055-9965.EPI-23-0038 PMC10233349

[20] Shavers VL . Measurement of socioeconomic status in health disparities research. J Natl Med Assoc. 2007;99:1013. 17913111 PMC2575866

[21] Cederberg M Hartsmar N Lingärde S . Thematic report: Socioeconomic disadvantage. Report from the EPASI (Educational Policies that Address Social Inequality) project supported by the European Commission’s department of Education & Culture, SOCRATES programme; 2009;2.

[22] Texas Department of State Health Services. Texas cancer Registry, 2024. Accessed January 8, 2024. https://www.dshs.texas.gov/texas-cancer-registry

[23] Census. American Community Survey Information Guide. Accessed January 12, 2024. https://www.census.gov/content/dam/Census/programs-surveys/acs/about/ACS_Information_Guide.pdf

[24] Census. Geographic Areas Reference Manual, Chapter 10: Census Tracts and Block Numbering Areas. 1994. Accessed October 3, 2020. https://www2.census.gov/geo/pdfs/reference/GARM/Ch10GARM.pdf

[25] Massey D . The prodigal paradigm returns: Ecology comes back to sociology In: Booth A, Crouter AC, eds. Does It Take A Village?: Community Effects on Children, Adolescents, and Families. Psychology Press; 2001:41–48.

[26] Krieger N Waterman PD Spasojevic J Li W Maduro G Van Wye G . Public health monitoring of privilege and deprivation with the index of concentration at the extremes. Am J Public Health. 2016;106:256–263. 26691119 10.2105/AJPH.2015.302955 PMC4815605

[27] Singh GK . Area deprivation and widening inequalities in US mortality, 1969–1998. Am J Public Health. 2003;93:1137–1143. 12835199 10.2105/ajph.93.7.1137 PMC1447923

[28] Knighton AJ Savitz L Belnap T Stephenson B VanDerslice J . Introduction of an area deprivation index measuring patient socioeconomic status in an integrated health system: Implications for population health. eGEMs. 2016;4:9. 10.13063/2327-9214.1238 PMC5019337 27683670

[29] Lee Y-CA Cohet C Yang Y-C Stayner L Hashibe M Straif K . Meta-analysis of epidemiologic studies on cigarette smoking and liver cancer. Int J Epidemiol. 2009;38:1497–1511. 19720726 10.1093/ije/dyp280

[30] Sohn W Lee HW Lee S Lim JH Lee MW Park CH . Obesity and the risk of primary liver cancer: A systematic review and meta-analysis. Clin Mol Hepatol. 2021;27:157. 33238333 10.3350/cmh.2020.0176 PMC7820201

[31] Suh JK Lee J Lee J-H Shin S Tchoe HJ Kwon J-W . Risk factors for developing liver cancer in people with and without liver disease. PLoS One. 2018;13:e0206374. 30372481 10.1371/journal.pone.0206374 PMC6205612

[32] Hall JM Szurek SM Cho H Guo Y Gutter MS Khalil GE . Cancer disparities related to poverty and rurality for 22 top cancers in Florida. Prev Med Rep. 2022;29:101922. 35928594 10.1016/j.pmedr.2022.101922 PMC9344025

[33] Centers for Disease Control and Prevention. PLACES. Accessed December 10, 2024. https://www.cdc.gov/places

[34] Cromartie J . Rural-Urban Commuting Area (RUCA) Codes. USDA Economic Research Service; 2019.

[35] Cameron AC Trivedi PK . Regression Analysis of Count Data. Cambridge University Press; 2013;53.

[36] VanderWeele TJ . Mediation analysis: A practitioner’s guide. Annu Rev Public Health. 2016;37:17–32. 26653405 10.1146/annurev-publhealth-032315-021402

[37] Lord D . Modeling motor vehicle crashes using Poisson-gamma models: Examining the effects of low sample mean values and small sample size on the estimation of the fixed dispersion parameter. Accid Anal Prev. 2006;38:751–766. 16545328 10.1016/j.aap.2006.02.001

[38] Brewer CA . Basic mapping principles for visualizing cancer data using geographic information systems (GIS). Am J Prev Med. 2006;30:S25–S36. 16458787 10.1016/j.amepre.2005.09.007

[39] Slocum TA McMaster RM Kessler FC Howard HH Mc Master RB . Thematic cartography and geographic visualization. Prentice Hall: Upper Saddle River, NJ, USA, 2008.

[40] Ananth CV . Proportion mediated in a causal mediation analysis: How useful is this measure? BJOG: Int J Obstet Gynaecol. 2019;126:983. 10.1111/1471-0528.15691 30893492

[41] White K Haas JS Williams DR . Elucidating the role of place in health care disparities: The example of racial/ethnic residential segregation. Health Serv Res. 2012;47:1278–1299. 22515933 10.1111/j.1475-6773.2012.01410.x PMC3417310

[42] Dankwa-Mullan I Pérez-Stable EJ . Addressing health disparities is a place-based issue. Am J Public Health. 2016;106:637–639. 26959267 10.2105/AJPH.2016.303077 PMC4816016

[43] Ortiz AG Wiese D Sorice KA Nguyen M González ET Henry KA . Liver cancer incidence and area-level geographic disparities in Pennsylvania—A geo-additive approach. Int J Environ Res Public Health. 2020;17:7526. 33081168 10.3390/ijerph17207526 PMC7588924

[44] Danos D Leonardi C Gilliland A Shankar S Srivastava RK Simonsen N . Increased risk of hepatocellular carcinoma associated with neighborhood concentrated disadvantage. Front Oncol. 2018;8:375. 30254987 10.3389/fonc.2018.00375 PMC6141716

[45] Ratnapradipa KL Li T Hsieh M-C Tenner L Peters ES . Most deprived Louisiana census tracts have higher hepatocellular carcinoma incidence and worse survival. Front Oncol, 14:1331049. 10.3389/fonc.2024.1331049 PMC10878418 38380357

[46] Zhao J Han X Zheng Z Nogueira LM Nathan PC Lu A . Racial/ethnic disparities in childhood cancer survival in the United States: Mediation effects of health insurance coverage and area-level social deprivation. Am Soc Clin Oncol. 2019;37:143.

[47] Duru OK Harawa NT Kermah D Norris KC . Allostatic load burden and racial disparities in mortality. J Natl Med Assoc. 2012;104:89–95. 22708252 10.1016/s0027-9684(15)30120-6 PMC3417124

[48] Geronimus AT . The weathering hypothesis and the health of African-American women and infants: Evidence and speculations. Ethn Dis. 1992;2:207–221. 1467758

[49] Tomfohr LM Pung MA Dimsdale JE . Mediators of the relationship between race and allostatic load in African and White Americans. Health Psychol. 2016;35:322. 27018723 10.1037/hea0000251

[50] Texas Cancer Registry, Cancer Epidemiology and Surveillance Branch . Cancer in Texas 2023. Texas Department of State Health Services; 2023.

