# Supplementary material for: Neighborhood-level deprivation mediates racial and ethnic disparities in HCC diagnosis in Texas
Source: Hepatol Commun. 2024 Oct 17;8(11):e0536. doi: 10.1097/HC9.0000000000000536 (PMC11495745; doi:10.1097/HC9.0000000000000536)
Supplement: Supplementary file 1 [file hc9-8-e0536-s001.docx]

**Neighborhood-level deprivation mediates racial and ethnic disparities in hepatocellular carcinoma diagnosis in Texas.**

**Authors:** Itunu O. Sokale,^1^ Aaron P. Thrift,^1,2^ Hashem B. El-Serag,^2,3,4,5^ Abiodun O. Oluyomi,^1,2,6*^

1. Section of Epidemiology and Population Sciences, Department of Medicine, Baylor College of Medicine, Houston, TX, USA
2. Dan L Duncan Comprehensive Cancer Center, Baylor College of Medicine, Houston, Texas, USA
3. Section of Gastroenterology and Hepatology and Clinical Epidemiology and Comparative Effectiveness Program in the Health Services Research, Michael E. DeBakey VA Medical Center, Baylor College of Medicine, Houston, TX, USA.
4. Clinical Epidemiology and Comparative Effectiveness Program, Section of Health Services Research (IQuESt), Michael E. DeBakey VA Medical Center, Baylor College of Medicine, Houston, TX, USA
5. Texas Medical Center Digestive Disease Center, Houston, TX, USA
6. Center for Precision Environmental Health, Baylor College of Medicine, Houston, TX, USA

SUPPLEMENTARY MATERIAL

**Index of Concentration at the Extreme (ICE) Calculation**

For our analysis, we calculated Black ICE and Hispanic ICE. For Black ICE, a value of -1 signifies the highest concentration of non-Hispanic Black individuals inside a census tract (i.e., non-Hispanic Black-concentrated tracts). For Hispanic ICE, -1 signifies the highest concentration of Hispanic individuals inside a census tract (i.e., Hispanic-concentrated tracts). For either Black ICE or Hispanic ICE, a value of 1 represents the highest concentration of non-Hispanic White individuals inside a census tract (i.e., non-Hispanic White-concentrated tracts).^1^ For the mediation analysis, the raw ICE scores were reclassified into a decile classification such that the most non-Hispanic White-concentrated tracts were in decile 1 while the most non-Hispanic Black- or Hispanic-concentrated tracts were in decile 10. Data to compute the ICE measures were the total number of Hispanic, non-Hispanic Black, and non-Hispanic White persons in each census tract. Of note, persons of other racial/ethnic identities were excluded from the calculations. Data were retrieved from the 2016-2020 ACS 5-year Estimates.

Reference

Krieger N, Waterman PD, Spasojevic J, Li W, Maduro G, Van Wye G. Public health monitoring of privilege and deprivation with the index of concentration at the extremes. *American journal of public health*. 2016;106(2):256-263.
